# The Association of Socio-Demographic Status, Lifestyle Factors and Dietary Patterns with Total Urinary Phthalates in Australian Men

**DOI:** 10.1371/journal.pone.0122140

**Published:** 2015-04-15

**Authors:** Peter Y. Bai, Gary A. Wittert, Anne W. Taylor, Sean A. Martin, Robert W. Milne, Zumin Shi

**Affiliations:** 1 Discipline of Medicine, University of Adelaide, Adelaide, South Australia, Australia; 2 School of Pharmacy and Medical Sciences, University of South Australia, Adelaide, South Australia, Australia; Universität Bochum, GERMANY

## Abstract

**Objective:**

To investigate the associations between socio-demographic status, lifestyle factors, dietary patterns and urinary total phthalate concentration in a cohort of South Australian men.

**Method:**

We randomly selected 1527 males aged 39 to 84 from wave two of the Men Androgen Inflammation Lifestyle Environment and Stress (MAILES) study. Total phthalate concentration was examined in fasting morning urine samples. Socio-demographic and lifestyle factors were assessed by questionnaire. Food intake was assessed by food frequency questionnaire (FFQ). Dietary patterns were constructed using factor analysis.

**Results:**

Total phthalates were detected in 99.6% of the urine samples. The overall geometric mean (95% CI) of total phthalate concentration was 112.4 (107.5–117.5) ng/mL. The least square geometric means (LSGMs) of total phthalate concentration were significantly higher among people who were obese (127.8 ng/mL), consuming less than two serves fruit per day (125.7 ng/mL) and drinking more than one can (375mL) of carbonated soft drink per day (131.9 ng/mL). Two dietary patterns were identified: a prudent dietary pattern and a western dietary pattern. Both the western dietary pattern (p = 0.002) and multiple lifestyle risk factors including smoking, obesity, insufficient physical activity and the highest quartile of the western dietary pattern (p<0.001), were positively associated with total phthalate levels. There was no significant relationship between total phthalate concentration and socio-demographic status.

**Conclusion:**

Phthalate exposure is ubiquitous and positively associated with lifestyle risk factors in urban dwelling Australian men.

## Introduction

Public concern has been raised about Phthalates due to ubiquitous exposure and concern regarding an association with detrimental health effects such as obesity, diabetes and cardiovascular disease. Phthalates are diesters of 1,2-benzenedicarboxylic acid (phthalic acid) [1] widely used in a variety of industrial and consumer products to increase the transparency, flexibility and durability of plastic [2]. They are also used as a solvent in personal care products, medications and dietary supplements [3, 4]. When used as a plasticiser, phthalates are not physically bonded to the material backbone resulting in their leaching [5] and, consequently, their detection in environmental samples such as surface water, soil and air [6], from where they may enter the human food chain [6].

The contamination of foods with phthalates may also occur from food packaging, food processing, storage and transport [7]. The concentrations of phthalates vary according to the nature of the food; levels are higher in foods with a greater fat content [5, 8, 9]. A high level of phthalates is found in spicy dressings and butter in Germany [8], fish products in the United Kingdom (UK) [5] and pork products in the United States (US) [9]. Phthalate exposure appears to be driven by dietary habit [10]. In an Italian study, high levels of certain phthalates were found in people consuming canned food, drinking alcohol and using plastic containers to store fatty foods [11].

Phthalates from contaminated food are absorbed via the gut, and diet is considered to be the major pathway of phthalate exposure [8]. Phthalates can also be absorbed by inhalation of contaminated air or dust, and through the skin following the use of personal care products. The contributions of these sources to phthalate exposure are considered to be minor [12–14]. The relationship between patterns of food intake and lifestyle factors and phthalate exposure has not, as far as we can tell, been reported.

Following absorption, most phthalates are rapidly metabolized to monoesters and excreted via the kidneys, appearing in the urine within 24–48 h [2, 15]. Some phthalates are stored in fat and can be found in human fluids beyond 48 h, which reflects the possibility of bioaccumulation [16, 17].

Some population-based studies have been undertaken to determine phthalate exposure levels. The Canadian Health Measures Survey (CHMS), which examined urine samples collected by mobile clinics from 2007 to 2009, detected 11 phthalate metabolites in more than 90% of Canadians [18]. Prior examination conducted by the National Health and Nutrition Examination Survey (NHANES) from 1999 to 2010 demonstrated the presence of phthalate metabolites in over 70% of Americans [19–21]. A study conducted in China in 2010 found that the presence of 14 phthalate metabolites in spot urine samples was ubiquitous [22].

Levels of phthalate metabolites are considered to vary according to socio-demographic status [19–21, 23–26]. However, the associations of phthalates and socio-demographic status are inconsistent in different populations, with no consistent pattern of change by socioeconomic status and ethnicity [21, 26]. Studies in Spain, China and central Italy considered that the regional variation might due to the level of industrial pollution, lifestyle and socioeconomic status developed in the region [11, 26, 27]. In a study comparing seven Asian countries, it was found that Kuwait had the highest level of phthalates compared to India, China, Vietnam, Japan, Korea, and Malaysia [28]. Similar studies have not, as far as we can establish, been undertaken in Australia, but are of potential importance given the health concerns attributed to phthalate exposure.

The aforementioned studies compared phthalate exposure by examining several phthalate metabolites. However, these findings may be biased by possible over-exposure to specific phthalates for a short period. In this study, we measured total phthalate levels in fasting morning urine samples collected from an urban, community-based cohort of South Australian men, to investigate the association of socio-demographic status, lifestyle factors and dietary patterns with total urinary phthalate concentration.

## Methods

### Study design and participants

This study used cross-sectional data from wave two of the Men Androgen Inflammation Lifestyle Environment and Stress (MAILES) study, a prospectively-followed cohort of urban, randomly selected community-dwelling men aged 35 and over at enrolment [29]. Fasting morning spot urine samples were available from men aged 39 to 84 who attended follow-up clinic visits and also completed detailed questionnaires to document socio-demographic status and health related behaviours (including food intakes), as well as health status and medication use (n = 1527 out of 2038 eligible participants of MAILES II). The study was approved by the research ethics committees of the Royal Adelaide Hospital and the North Western Adelaide Health Service, and informed consent was obtained from all participants. A written informed consent form was given to the eligible participants who signed in-clinic.

### Measurements of phthalates

First-morning urine samples were collected in the clinic after an overnight fast, in 125 mL Nalgene containers (Thermo-Fisher Scientific Inc., Rochester NY, USA), aliquot into 30 mL Nalgene containers, stored on ice up to 2 h post-collection before being stored at -80°C. Total phthalates were measured using liquid chromatography tandem mass spectrometry (LC-MS/MS). The method extracts phthalic acid (PA) from participant urine samples (total PA, after hydrolysis of the glucuronides with 3 mol/L HCl), calibration standards (containing ^13^C_4_-PA, added as an external calibration standard and ^2^H_4_-PA, added as internal standard to all samples) and quality controls (containing ^13^C_4_-PA and ^2^H_4_-PA) using solid phase extraction (Strata-X polymeric reversed-phase, Phenomenex, Lane Cove, NSW, Australia). The analytes were separated by HPLC on a phenyl hexyl column (Luna, 50 x 2.0 mm, 3 μ, Phenomenex, Lane Cove, NSW, Australia) preceded by a 2 mm cartridge of the same material, and the eluate monitored with an API3000 MS/MS (AB Sciex, Mulgrave, Vic, Australia) operating in negative Multiple Reaction Monitoring (MRM) mode. The singly-charged Q1/Q3 transition for PA is 165.0/121.0/77.0 atomic mass units (amu), for ^13^C_4_-PA is 169.1/124.0/79.0 and 169.0/124.0/79.0 amu for ^2^H_4_-PA. The concentration of PA was calculated from a calibration curve of the ratio of the peak area of ^13^C_4_-PA/^2^H_4_-PA versus concentrations of ^13^C_4_-PA. The data were acquired and processed with Analyst 1.4 linked directly to the mass spectrometer. The assay range was from 10 to 1500 ng/mL and had a run time of approximately 10 min per sample at a flow rate of 0.2 mL/min. To prevent contamination from plastics during processing, the connection with plastic lids or containers was minimized. For concentrations of total phthalates (n = 6) below the limit of quantification (LOQ) of 10 ng/ml, a value of the LOQ divided by two was given [18].

### Dietary patterns

Dietary intakes were assessed by a validated food frequency questionnaire (FFQ) [30]. Participants (n = 1503) who have completed FFQ reported the frequency that particular food items were consumed in the previous 12 months (never, monthly, weekly and daily). The frequencies of monthly and weekly food consumption were converted to daily frequencies using daily equivalent coefficients [30]. A total number of 128 food items including alcohol (beer, wine and spirit) and beverages (carbonated soft drink, tea and coffee) were aggregated into 40 groups based on a previous study in Australia (S1 Table) [30]. The 40 food groups were used to construct dietary patterns through factor analysis. There were a number of missing data for the daily consumption of fruit (n = 4), vegetables (n = 4), soft drinks (n = 4) and alcohol (n = 2). These were assigned a value of zero when the dietary patterns were constructed. Factor analysis was performed using the principle component factor method. The numbers of factors were retained in factor analysis by considering an Eigenvalue that was greater than one and a Scree plot. We tried two to five factor solutions. Two factors were finally used in the factor analysis according to the interpretability of derived patterns. Factors obtained were rotated using the (orthogonal) Varimax procedure to facilitate interpretability and ensure orthogonality [30]. Factor loadings were plotted in graphs in order to visualize the composition of food patterns. The major contributors of the dietary patterns was considered as food items with factor loadings greater than 0.3. The scores were predicted for both derived dietary patterns (prudent dietary and western dietary) and divided into quartiles for further analyses.

### Socio-demographic and lifestyle factors

The participants’ socio-demographic data (including age, marital status, employment status, the highest educational level and annual household income), smoking status and physical activity by Active Australia survey definition [31] were collected using a standardised self-reported questionnaire [29]. Socio-demographic status including education was categorized according to the 13^th^ biennial report of the Australian Institute of Health and Welfare (AIHW) [32]. The sufficient physical activity was defined as undertaking 150 mins or more of activity per week [31]. Body mass index (BMI) was calculated from weight (kg) divided by the square of height (m) with weight and height measured without shoes and in light clothing at the clinic [29]. A multiple lifestyle risk factors model was constructed based on smoking, obesity, undertaking insufficient physical activity and the highest quartile of western dietary pattern (each scored 1).

### Occupational exposure

Participants were asked if they ever worked in a range of industries which were potentially exposed to hazardous substance. The listed industries include plastic fumes/plastic industry, cleaning agents industry, solvents/paints/remover industry, benzene industry, anaesthetics industry, printing ink industry, glue industry, wood dust/mining industry, asbestos industry, diesel fumes industry, lead industry, soldering fumes industry, pesticide/crop spraying industry, other chemical industry (not specified), other fumes industry (not specified). One more question was also asked to clarify if people are currently working in those industries. The occupational exposure status was created by categorizing the participants into three groups including none, ex-workers and current workers.

### Statistical analysis

As the total urinary phthalate concentration were log-normal distributed, the geometric mean (GM) was calculated to represent overall phthalate exposure. Two multiple linear regression models were constructed. To compare the difference of the GM of total phthalate concentration across socio-demographic status and lifestyle factors, a multivariable linear regression model was produced to obtain the mutually adjusted least square geometric means (LSGMs) and 95% confidence intervals (CIs) of total phthalates concentration. The model includes socio-demographic status such as age, education, employment status, marital status, household annual income, and lifestyle factors such as smoking, BMI, physical activity, and daily consumption of alcohol, fruit, vegetables and carbonated soft drink [5, 18, 33]. The LSGMs were obtained through margins command with exponential option [34]. The pairwise comparisons across levels of factor variables were conducted after the regression. Hierarchical linear regression models were built to estimate the associations of total phthalate concentration with quartiles of dietary patterns and multiple lifestyle risk factors by adjusting for a broad range of known and potential confounders [20, 35]. We built three models: model 1 adjusted for age; level 2 with additional adjustment for the remained socio-demographic status including education, employment status, marital status and annual household income; level 3 further adjusted for the lifestyle factors including current smoking, BMI and physical activity for dietary patterns. Wald test was performed to compare the regression coefficients among groups. A sensitivity test was conducted to assess the effect of occupational exposure. A *p*-value ≤ 0.05 was considered to be significant. All analyses were performed and graphs drawn using STATA version 12 (StataCorp, College Station, TX, USA).

## Results

The sample characteristics are shown in Table 1. The mean age of participants was 59.6 years. Among all men, 46.7% had a diploma or higher education attainment; 48.1% worked full-time and more than one third (35.5%) were retired. Over 60% of the participants reported that their gross annual household income was greater than AU$40,000. The majority of the participants in the study were overweight or obese (81.3%), alcohol drinkers (88.9%) and consumed fewer than five serves of vegetables per day (95.9%). There were 14.6% and 15.3% of total participants who were current smokers and drinking more than one can of carbonated soft drink per day respectively.

**Table 1:** Basic sample characteristic.

	**n (%)**
**Overall**	1527 (100.0)
**Education** ^a^	
Up to high school	426 (28.2)
Trade/apprenticeship	380 (25.1)
Certificate/diploma	487 (32.2)
Degree or higher	220 (14.5)
**Employment** ^a^	
Full time	717 (48.1)
Part time/unemployed/student/other	243 (16.3)
Retired	529 (35.5)
**Marital status** ^a^	
Married/living with a partner	1169 (78.7)
Separated/divorced/widowed	225 (15.2)
Never married	91 (6.1)
**Household annual income** ^a^	
Up to $40,000	531 (37.0)
$40,001 to $80,000	504 (35.2)
$80,001 or more	399 (27.8)
**Smoking** ^a^	
Non/ex-smokers	1285 (85.4)
Current smokers	219 (14.6)
**Body Mass Index (BMI)** ^a^	
Underweight/normal (< = 24.9 kg/m^2^)	285 (18.7)
Overweight (25.0–29.9 kg/m^2^)	723 (47.4)
Obesity (> = 30.0 kg/m^2^)	518 (33.9)
**Physical activity** ^a^	
Sufficient physical activity	597 (60.6)
Insufficient physical activity	388 (39.4)
**Alcohol per day** ^a^ ^b^	
Non alcohol drinkers	166 (11.1)
Alcohol drinkers	1335 (88.9)
**Fruit per day** ^a^ ^b^	
2 serves or more	609 (40.6)
Less than 2 serves	890 (59.4)
**Vegetables per day** ^a^ ^b^	
5 serves or more	62 (4.1)
Less than 5 serves	1437 (95.9)
**Carbonated soft drinks per day** ^a^ ^b^	
1 can or less	1270 (84.7)
More than 1 can	229 (15.3)

^a^ Missing categories were not reported.

^b^ Questions from food frequency questionnaire (FFQ).

**Fig 1:**
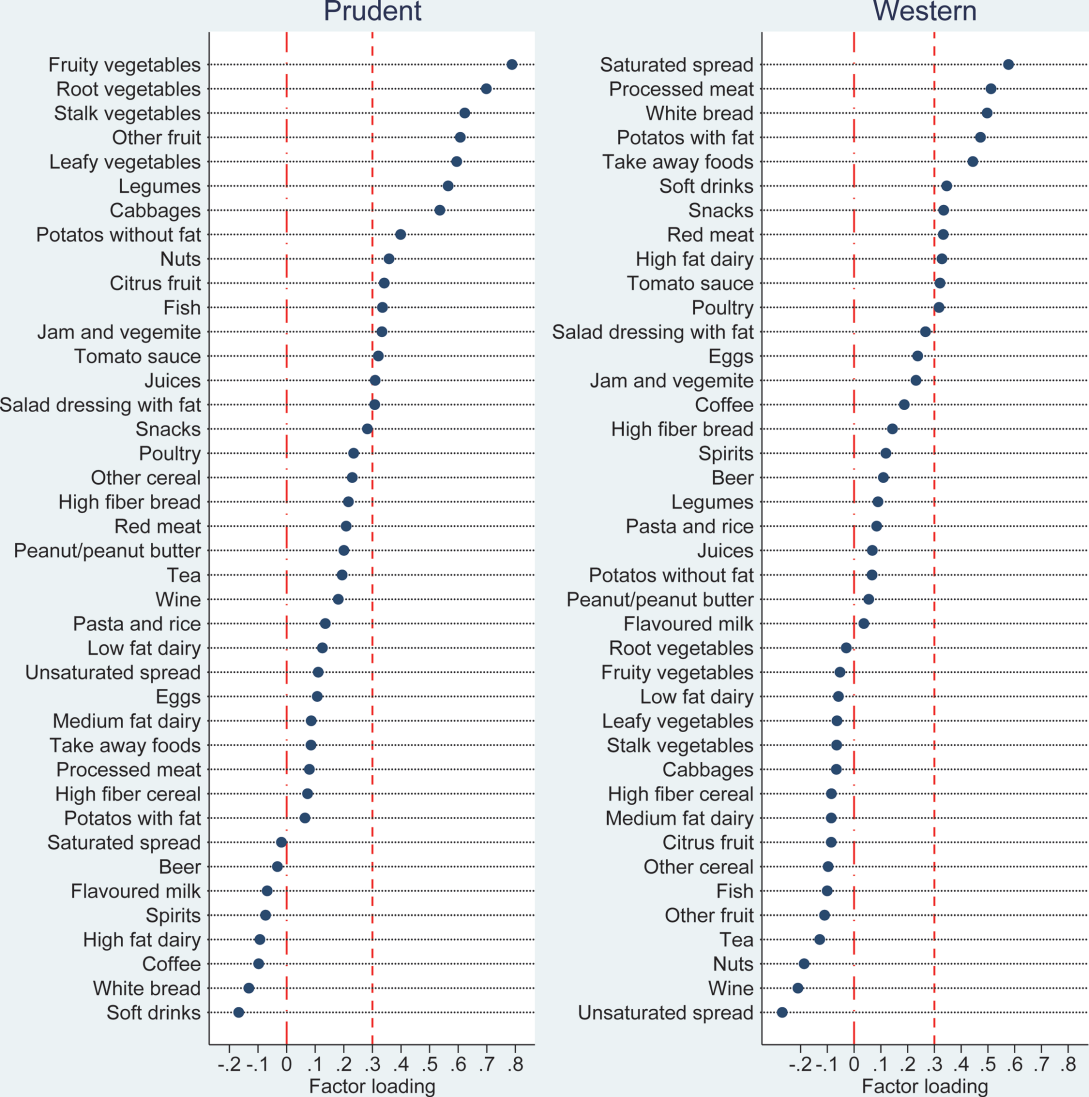
The factor loadings of factor analysis for dietary patterns. Two dietary patterns which are prudent and western dietary pattern were generated using the principle component factor method of factor analysis.

Two dietary patterns were identified and the labelled rotated factor loadings are plotted in Fig. 1. The two dietary patterns accounted for 16.9% of the total variance of food intake. Factor 1 was named as the prudent dietary pattern as it had high factor loadings on fruit, vegetables, fish, legumes, nuts and juices. Factor 2 was named as the western dietary pattern as it had high factor loadings on high fat dairy, take-away food, processed meat, carbonated soft drink, white bread, saturated spread and potato cooked with fat.

**Fig 2:**
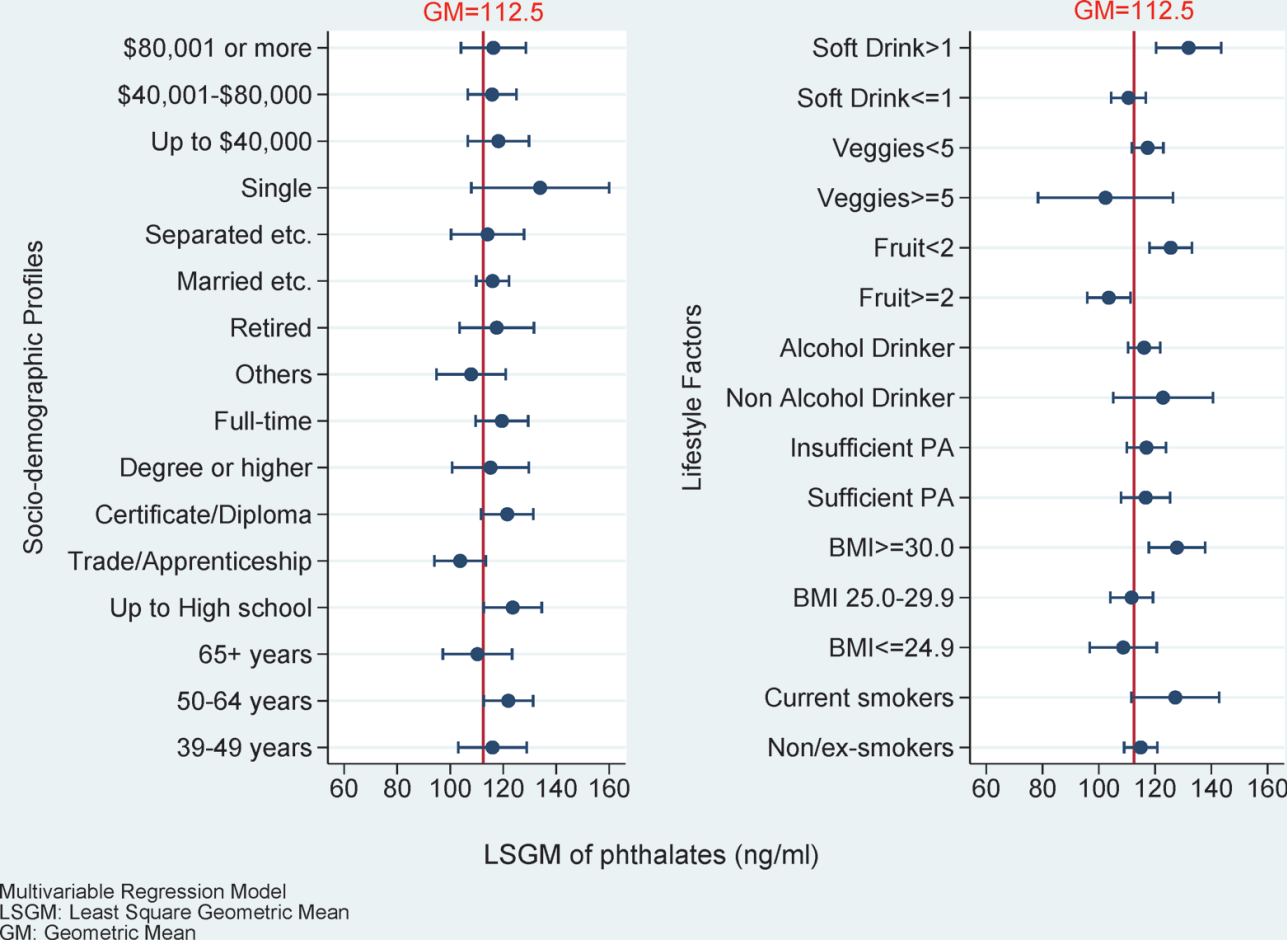
The LSGM of phthalate concentration among socio-demographic status and lifestyle factors. Margins with exponential transferring and marginsplot syntax were used to plot after linear regression mutually adjusting for age, education, employment status, marital status, household annual income, smoking, body mass index (BMI), physical activity, alcohol consumption per day, fruit consumption per day, vegetables consumption per day and carbonated soft drinks consumption per day.

Total phthalates were detected in 99.6% of the urine samples. The overall GM (95% CI) of total phthalate concentration was 112.4 (107.5–117.5) ng/mL (S2 Table). The adjusted LGSMs of total phthalate concentration among socio-demographic status and lifestyle factors are plotted in Fig. 2. There were no significant differences in total phthalate concentration among socio-demographic status. However, the LGSM of total phthalate concentration was significantly higher among obese men (127.8 ng/mL vs 108.8 ng/mL, *p* = 0.019), as well as those consuming less than two serves of fruit (125.7 ng/mL vs 103.6 ng/mL, *p*<0.001) and drinking more than one can (375 mL) of carbonated soft drink, per day (131.9 ng/mL vs 110.7 ng/mL, *p* = 0.001).

The associations of total phthalate concentration with dietary patterns and multiple lifestyle risk factors are shown in Tables 2 and 3. The western dietary pattern was positively associated with the LSGM of the urine phthalate concentration (*p* = 0.002), of which increased by 18.7% in-group with the highest quartile of western dietary pattern compared to the lowest quartile (125.7 ng/mL vs 105.9 ng/mL). A marginally inversed association was found in those with the prudent dietary pattern (*p* = 0.043). Nearly 80% of people had at least one lifestyle risk factor. The phthalate concentration increased with the number of lifestyle risk factors (*p*<0.001); people with three or more lifestyle risk factors had 22.8% higher concentrations than those who had no lifestyle risk factors.

**Table 2:** The association between dietary patterns and urinary total phthalate concentration.

	**n**	**LSGM** ^a^ ^d^ **(95% CI)**	β1 **(SE)** ^b^	β2 **(SE)** ^c^	β3 **(SE)** ^d^
**Prudent dietary pattern** ^e^					
Quartile 1 (lowest)	376	120.7 (109.4–132.1)	0 (ref)	0 (ref)	0 (ref)
Quartile 2	376	124.5 (113.1–135.9)	0.02 (0.06)	0.03 (0.07)	0.03 (0.07)
Quartile 3	376	112.8 (102.3–123.3)	-0.10 (0.06)	-0.08 (0.07)	-0.07 (0.07)
Quartile 4 (highest)	375	107.4 (97.3–117.4)	-0.12 (0.06)	-0.12 (0.07)	-0.11 (0.07)
*P* value for trend			**0.024**	**0.028**	**0.047**
**Western dietary pattern** ^e^					
Quartile 1 (lowest)	376	105.9 (95.8–115.9)	0 (ref)	0 (ref)	0 (ref)
Quartile 2	376	108.3 (98.2–118.3)	0.07 (0.06)	0.02 (0.07)	0.02 (0.07)
Quartile 3	376	125.3 (113.9–136.8)	0.21 (0.06)	0.17 (0.07)	0.17 (0.07)
Quartile 4 (highest)	375	125.7 (113.9–137.6)	0.23 (0.06)	0.20 (0.07)	0.17 (0.07)
*P* value for trend			**<0.001**	**<0.001**	**0.002**

^a^ LSGM: Least Square Geometric Mean.

^b^ Model adjusted to age.

^c^ Model adjusted to age, education, employment status, marital status and annual household income.

^d^ Model adjusted to age, education, employment status, marital status, annual household income, current smoking, body mass index (BMI) and physical activity.

^e^ Derived from food frequency questionnaire (FFQ).

**Table 3:** The association of a multiple lifestyle risk factors with urinary total phthalate concentration.

	**n**	**LSGM** ^a^ ^c^ **(95% CI)**	β1 **(SE)** ^b^	β2 **(SE)** ^c^
**Lifestyle risk factors** ^d^				
None	311	103.9 (93.3–114.5)	0 (ref)	0 (ref)
1 risk factor	599	108.0 (100.0–115.9)	0.03 (0.06)	0.04 (0.06)
2 risk factors	431	130.2 (118.9–141.4)	0.23 (0.07)	0.23 (0.07)
3 factors or more	186	127.6 (111.0–144.3)	0.29 (0.08)	0.21 (0.09)
*P* value for trend			**<0.001**	**<0.001**

^a^ LSGM: Least Square Geometric Mean.

^b^ Model adjusted to age.

^c^ Model adjusted to age, education, employment status, marital status and annual household income.

^d^ Multiple lifestyle risk factors include smoking, obesity, insufficient physical activity and highest quartile of western dietary pattern.

We conducted a sensitivity analysis to test the effect of occupational exposure (S3 Table). After adjusted for age, remained socio-demographic status and lifestyle factors respectively, there was no significant association between occupational exposure and phthalate levels.

## Discussion

In this cross-sectional study we detected the presence of total phthalates in the urine of almost all participants. This indicates that at least for urban dwelling South Australian men there is ubiquitous exposure to phthalates. The positive association of a western dietary pattern, higher carbonated soft drink consumption, lower fruit consumption and obesity with total phthalate concentration suggests that lifestyle factors and chronic disease risk are co-associated with phthalate exposure.

Although previous studies [18, 19, 24, 26] have shown almost universal exposure to phthalates, as far as we are aware, this is the first population study to explore the relationship between urinary phthalate concentration, dietary patterns and lifestyle risk factors. It is also the first study of phthalate exposure in an Australian population. In Australia, people with unhealthy lifestyles tend to consume more fast or convenience foods [36]. As dietary intake is the primary route of phthalate exposure [37], it seems most likely that phthalate exposure result from an unhealthy dietary pattern of processed and packaged foods [5, 7–9] often associated with other unhealthy behaviours and obesity [38, 39]. The association of carbonated soft drink consumption with phthalate exposure has been reported previously [40]. Most carbonated soft drinks are consumed from containers either made of, or lined with, material that can leach phthalates into the fluid [41]. It is also possible that the contamination occurs during processing [42].

In contrast The inverse association between the prudent dietary pattern and urinary phthalate reflects the consumption of more fresh vegetables, fruit, nuts and fish and less high fat, packaged and processed foods [5], and consequently less exposure to phthalates.

The higher urinary phthalate in obese men may reflect patterns of food intake, but since the observation is independent of diet it is more likely to be the result of phthalate accumulation in adipose tissue. A recent study in rats has shown that dibutyl phthalate (DBP) accumulates in adipose tissues [17].

The absence of an association between age and total phthalate concentration is in accordance with other studies [18, 24, 25]. There was also no association found in this study between urinary total phthalate concentration and socioeconomic status, however, this differs from the findings of previous studies [19–21, 23, 26, 43–46]. Firstly, this may be because we examined total phthalates rather than specific phthalate metabolites which were measured in all of previous studies. Secondly, some of these studies only recruited maternal women or women at reproductive age, which are different from our target population [20, 21, 26, 43–46]. Thirdly, results from the studies using NHANES data showed an inconsistent pattern over time [19–21, 23]. Finally, the association between the level of some phthalate metabolites and education, household income and social class remain in conflict among a number of studies on women [20, 21, 26, 43–46]. Therefore, our findings among socioeconomic status are incomparable with previous studies due to different study designs, different target population and different regions. It is surprising that occupational exposure did not affect the total phthalate concentration in this study. They may due to a good occupational protection or that participants were absent from work 24–48 h before the urine collection. However, further research may need to investigate this association.

The strength of this study is the measurement of total urinary phthalates from a large sample of community dwelling men from whom detailed information relating to socio-demographic status, lifestyle and diet was obtained. Total phthalate concentration is less subject to variation as compared to specific phthalate metabolites measured in previous studies [20], and is a good indicator of the overall impact exposure to multiple sources of environmental phthalates [47–49].

The limitations of this study include its cross-sectional nature and so causal relationships between lifestyle factors and total phthalate exposure cannot be established. Also while the men in the study are broadly representative of Australian urban dwelling men aged 39–84 years, the findings cannot be generalised beyond this group. In addition, we did not have detailed information on smoking. We were unable to adjust for the day-to-day variation in phthalate exposure, although this may not affect the overall direction of the associations. Finally, we did not collect contemporaneous dietary records and therefore we are relying on the “usual dietary pattern” rather than exposure over the previous 24 hours.

## Conclusion

As the first study to explore the total phthalate exposure among Australians, we found that phthalates are ubiquitous. Lifestyle factors and dietary habits contribute more to phthalate exposure than socio-demographic status. This extends our understanding on the determinants of total phthalate exposure in Australia. Our findings also support that dietary intake is a major route of phthalate exposure, with carbonated soft drink being an important contributor. People consuming more fatty foods are more likely to have a high concentration of phthalates in the body. Therefore, adopting a healthy lifestyle may be an option to reduce phthalate exposure. Given the ubiquitous but markedly varying exposure to phthalates future research is needed to explore the relationships between total phthalate exposure and health risks such as obesity, diabetes and cardiovascular disease [1].

## Supporting Information

S1 Table Composition of 40 food groups used for dietary patterns analysis. (DOCX)

S2 Table The distribution of total phthalates (ng/mL) in South Australian men. (DOCX)

S3 Table The sensitivity test for occupational exposure and total phthalates. (DOCX)
