# Supplementary material for: The Association of Socio-Demographic Status, Lifestyle Factors and Dietary Patterns with Total Urinary Phthalates in Australian Men
Source: PLoS One. 2015 Apr 15;10(4):e0122140. doi: 10.1371/journal.pone.0122140 (PMC4398403; doi:10.1371/journal.pone.0122140)
Supplement: S1 Table — (DOCX) [file pone.0122140.s001.docx]

**Table S1: Composition of 40 food groups used for dietary patterns analysis**

| **Food groups** | **Food items** |
| --- | --- |
| High fat dairy | Full cream milk, other cheese, cream or sour cream |
| Medium fat dairy | Reduced fat milk, soy milk, ricotta or cottage cheese, yogurt |
| Low fat dairy | Skim milk, low calorie/fat dressing |
| Flavoured milk | Flavoured milk |
| Juices | Orange juice, other fruit juice, canned tomatoes and tomato juice |
| Soft drinks | Diet soft drink, regular soft drink |
| Tea | Tea, herbal tea |
| Coffee | Coffee, coffee substitute |
| Red meat | Beef or veal, lamb, pork |
| Processed meat | Sausages, processed meat, bacon |
| Poultry | Chicken |
| Fish | Steamed/grilled/baked fish, tinned fish |
| Take away foods | Fried fish, pastries with cheese, pastries with meat, pizza |
| Pasta and rice | Rice, pasta or noodles |
| High fibre cereal | All-Bran, bran or hi fibre cereal, Sultana bran |
| Other cereal | Corn flakes, fibre plus, health wise, just right, muesli (any variety), nutri grain, rice bubbles/puffed rice, special k, sustain, sports plus, vita brits, weet bix regular/other, weeties, porridge, breakfast cereals |
| Potatoes with fat | Potato roasted or fried |
| Potatoes without fat | Potato steamed, boiled or baked |
| Citrus fruit | Orange or other citrus fruit |
| Other fruit | All other fruits including tinned fruit |
| Fruity vegetables | Fresh tomatoes, capsicum, avocado, cucumber, mushroom, pumpkin, zucchini, eggplant, green beans, olives |
| Stalk vegetables | Asparagus, onion/leeks, celery, garlic |
| Root vegetables | Sweet potato, beetroot, carrot |
| Cabbages | Brussels sprouts, cauliflower, broccoli |
| Leafy vegetables | Lettuce, salad leaves, Asian greens, other cooked leaves, coleslaw |
| Legumes | Green peas, sweet corn, baked beans, dried beans/peas |
| High fibre bread | High fibre white, wholemeal, multi-grain, rye, soy and linseed |
| White bread | White bread |
| Eggs | Egg |
| Unsaturated spread | Becel, logicol, nuttelex, olive oil, omega, pro-active, soy, sterol |
| Saturated spread | Butter, margarine |
| Beer | Beer |
| Wine | Wine |
| Spirits | Spirit |
| Snacks | Cracker, biscuits, cakes, ice-cream, confectionery, chips |
| Other nuts | Other nuts |
| Peanuts/peanut butter | Peanuts, peanut butter |
| Jam and vegemite | Jam, vegemite |
| Tomato sauce | Tomato puree, tomato sauce, ketchup |
| Salad dressing with fat | Margarine/butter/oil on cooked vegetables, mayonnaise, oil and vinegar salad dressing |
