# Supplementary material for: The Association of Socio-Demographic Status, Lifestyle Factors and Dietary Patterns with Total Urinary Phthalates in Australian Men
Source: PLoS One. 2015 Apr 15;10(4):e0122140. doi: 10.1371/journal.pone.0122140 (PMC4398403; doi:10.1371/journal.pone.0122140)
Supplement: S2 Table — (DOCX) [file pone.0122140.s002.docx]

**Table S2: The distribution of total phthalates (ng/mL) in South Australian men**

|  | **Rang** | **GM** | **Mean** | **25th** | **50th** | **75th** |
| --- | --- | --- | --- | --- | --- | --- |
| **Overall** | 5-997 | 112.4 | 164.0 | 65.0 | 114.0 | 197.0 |
| **Education** |  |  |  |  |  |  |
| Up to high school | 11-997 | 122.3 | 182.3 | 69.0 | 118.5 | 225.0 |
| Trade/apprenticeship | 5-906 | 100.7 | 147.1 | 60.5 | 105.0 | 161.5 |
| Certificate/diploma | 5-945 | 116.7 | 168.0 | 67.0 | 119.0 | 199.0 |
| Degree or higher | 10-718 | 107.6 | 150.0 | 65.0 | 106.5 | 204.5 |
| **Employment** |  |  |  |  |  |  |
| Full time | 5-925 | 116.6 | 168.2 | 69.0 | 122.0 | 206.0 |
| Part time/unemployed/student/other | 13-997 | 113.1 | 159.8 | 67.0 | 112.0 | 194.0 |
| Retired | 5-986 | 109.6 | 164.1 | 63.0 | 107.0 | 180.0 |
| **Marital status** |  |  |  |  |  |  |
| Married/living with a partner | 5-997 | 112.3 | 163.4 | 65.0 | 113.0 | 196.0 |
| Separated/divorced/widowed | 5-986 | 112.9 | 164.2 | 66.0 | 120.0 | 195.0 |
| Never married | 19-963 | 132.4 | 194.3 | 71.0 | 125.0 | 235.0 |
| **Household annual income** |  |  |  |  |  |  |
| Up to $40,000 | 5-986 | 113.6 | 169.2 | 64.0 | 109.0 | 190.0 |
| $40,001 to $80,000 | 5-997 | 114.1 | 164.9 | 67.0 | 117.5 | 199.5 |
| $80,001 or more | 5-925 | 115.2 | 165.0 | 69.0 | 122.0 | 198.0 |
| **Smoking** |  |  |  |  |  |  |
| Non/ex-smokers | 5-997 | 109.5 | 161.0 | 63.0 | 111.0 | 192.0 |
| Current smokers | 5-865 | 132.2 | 181.8 | 83.0 | 129.0 | 243.0 |
| **Body Mass Index (BMI)** |  |  |  |  |  |  |
| Underweight/normal (<25) | 5-876 | 103.0 | 149.6 | 60.0 | 103.0 | 175.0 |
| Overweight (25-29) | 5-997 | 107.0 | 161.7 | 59.0 | 110.0 | 186.0 |
| Obesity (>29) | 9-925 | 125.9 | 173.6 | 76.0 | 128.5 | 215.0 |
| **Physical activity** |  |  |  |  |  |  |
| Sufficient physical activity | 5-997 | 109.6 | 164.2 | 62.0 | 110.0 | 201.0 |
| Insufficient physical activity | 5-976 | 114.2 | 163.8 | 67.0 | 115.5 | 194.0 |
| **Alcohol per day** |  |  |  |  |  |  |
| Non alcohol drinkers | 13-888 | 117.7 | 163.9 | 65.0 | 111.5 | 190.0 |
| Alcohol drinkers | 5-997 | 112.1 | 164.2 | 66.0 | 114.0 | 197.0 |
| **Fruit per day** |  |  |  |  |  |  |
| 2 serves or more | 5-997 | 97.9 | 145.3 | 57.0 | 98.0 | 169.0 |
| Less than 2 serves | 5-986 | 123.9 | 177.0 | 74.0 | 126.5 | 223.0 |
| **Vegetables per day** |  |  |  |  |  |  |
| 5 serves or more | 14-536 | 94.0 | 132.2 | 54.0 | 104.5 | 169.0 |
| Less than 5 serves | 5-997 | 113.5 | 165.5 | 66.0 | 114.0 | 198.0 |
| **Carbonated soft drinks per day** |  |  |  |  |  |  |
| 1 can or less | 5-986 | 105.4 | 155.4 | 61.0 | 107.0 | 184.0 |
| More than 1 can | 10-997 | 133.9 | 187.1 | 79.0 | 132.5 | 228.0 |
