# Supplementary material for: The Association of Socio-Demographic Status, Lifestyle Factors and Dietary Patterns with Total Urinary Phthalates in Australian Men
Source: PLoS One. 2015 Apr 15;10(4):e0122140. doi: 10.1371/journal.pone.0122140 (PMC4398403; doi:10.1371/journal.pone.0122140)
Supplement: S3 Table — (DOCX) [file pone.0122140.s003.docx]

**Table S3: The sensitivity test for occupational exposure ^e^ and total phthalates**

|  | **n** | **LSGM ^ad^ (95% CI)** | β1 **(SE) ^b^** | β2 **(SE) ^c^** | β3 **(SE) ^d^** |
| --- | --- | --- | --- | --- | --- |
| None | 303 | 129.4 (115.4-143.3) | 0 (ref) | 0 (ref) | 0 (ref) |
| Ex-workers | 689 | 111.9 (103.9-120.0) | -0.11 (0.06) | -0.12 (0.07) | -0.14 (0.07) |
| Current workers | 282 | 113.9 (100.8-127.0) | -0.09 (0.08) | -0.11 (0.08) | -0.13 (0.08) |
| *P* value for trend |  |  | 0.213 | 0.150 | 0.091 |

^a^ LSGM: Least Square Geometric Mean

^b^ Model adjusted to age

^c^ Model adjusted to age, education, employment status, marital status and annual household income

^d^ Model adjusted to age, education, employment status, marital status, annual household income, current smoking, body mass index (BMI), physical activity, prudent dietary patterns and western dietary patterns

^e^ Occupational exposure derived from occupations in plastic fumes/plastic industry, cleaning agents industry, solvents/paints/remover industry, benzene industry, anaesthetics industry, printing ink industry, glue industry, wood dust/mining industry, asbestos industry, diesel fumes industry, lead industry, soldering fumes industry, pesticide/crop spraying industry, other chemical industry (not specified), other fumes industry (not specified)
